# Antagonistic potential of Moroccan entomopathogenic nematodes against root-knot nematodes, *Meloidogyne javanica* on tomato under greenhouse conditions

**DOI:** 10.1038/s41598-022-07039-0

**Published:** 2022-02-21

**Authors:** Ali El Aimani, Abdellah Houari, Salah-Eddine Laasli, Rachid Mentag, Driss Iraqi, Ghizlane Diria, Slimane Khayi, Rachid Lahlali, Abdelfattah A. Dababat, Fouad Mokrini

**Affiliations:** 1 Taroudant Multidisciplinary Faculty, Laboratoire de Biotechnologie, Valorisation et Envirennement, Agadir, Morocco; 2 Laboratory of Botany, Mycology, and Environment, Faculty of Science, Mohammed V University, Rabat, Morocco; 3 Biotechnology Research Unit, Laboratory of Nematology, National Institute of Agricultural Research, INRA-Morocco, Rabat, Morocco; 4 Phytopathology Unit, Department of Plant Protection, Ecole Nationale d’Agriculture de Meknes, km. 10, Route Haj Kaddour, B.P. S/40, 50001 Meknes, Morocco; 5 International Maize and Wheat Improvement Center (CIMMYT), P.K. 39, Emek, 06511 Ankara, Turkey

**Keywords:** Plant sciences, Plant physiology, Plant stress responses

## Abstract

The root-knot nematode, *Meloidogyne javanica* is a devastating pest affecting tomato production worldwide. Entomopathogenic nematodes (EPNs) are considered very promising biocontrol agents that could be used to effectively manage plant-parasitic nematode. The antagonistic activity of five EPN strains isolated from different fields in Morocco was evaluated against juvenile (J2s) antagonism in soil, the number of egg masses, and the galling index of *M. javanica* and J2s reproduction in the root. In greenhouse experiments, *Steinernema feltiae* strains (EL45 and SF-MOR9), *Steinernema* sp. (EL30), and those of *Heterorhabditis bacteriophora* (HB-MOR7 and EL27) were applied to the soil alongside RKN J2s. There was a significant reduction in *M. javanica* densities in the soil and roots by EPNs treatments when compared to the positive control. The EPNs decreased both egg masses formation and galling index by 80% compared to the positive control. The application of EPNs at a rate of 50 and 75 infective juveniles (IJs) cm^−2^ gave significant control of all studied nematological parameters compared to the positive control, which confirmed the importance of the doses applied. The applied dose was significantly correlated with *M. javanica* parameters according to polynomial regression models. The results also showed that *S. feltiae* strain (EL45) significantly increased plant height and root length, while *H. bacteriophora* strain (HB-MOR7) only enhanced root fresh weight. Therefore, both indigenous EPN strains; EL45 and SF-MOR9 have eco-friendly biological potential against *M. javanica* in vegetable crops.

## Introduction

Plant-parasitic nematodes (PPNs) are viewed as major biotic constraints causing great damage and yield loss to the majority of crops worldwide^1–3^. They generate an outstanding global economic loss, estimated at $173 billion^4^. The root-knot nematode (RKN) of the genus *Meloidogyne* is one of the most devastating PPNs worldwide and comprises of more than 100 species^5^. Four species (*Meloidogyne javanica, M. incognita, M. hapla*, and *M. arenaria*) are polyphagous and common species that have the most damaging effects on vegetable crop production^6^. *Meloidogyne* spp. caused significant yield losses that could be reached up to 80% in tomato growing areas^7^. In Morocco, the RKNs are among the most relevant and damaging groups of PPNs and are widely distributed throughout the country^8,9^. *Meloidogyne incognita* and *M. javanica* have been reported to be the most common species^8,10^. This wide distribution and frequent occurrence of RKNs require effective management strategies to keep their damage under the threshold levels.

Entomopathogenic nematodes (EPNs) of the genera *Steinernema* (Panagrolaimomorpha: Steinernematidae) and *Heterorhabditis* (Rhabditomorpha: Heterorhabditidae) are potential biocontrol agents that showed efficient antagonistic effects against many insect pests^11^. These beneficial nematodes invade and kill insects within 24–48 h via the toxins released by their symbiotic bacteria of *Xenorhabdus* and *Photorhabdus* in *Steinernema* and *Heterorhabditis*, respectively, into their hemocoel^12–14^. Bacteria produce some apoptosis or necrosis-induced substances (e.g., hemolysin, cytolysin, and toxins) in the host cells which trigger its death^15^.

Management of PPNs using biological control agents (BCAs) is a promising alternative to the chemical alternatives^16,17^. The antagonistic activity exerted by EPNs on PPNs has been previously observed and reported^18^. The management of EPNs against different nematode species, such as *Criconemoides* spp., *Rotylenchulus reniformis*^19^, *Globodera rostochiensis*^20^, *Belonolaimus longicaudatus*^21^, and *Meloidogyne* spp.^22–25^ has been proven under both field and greenhouse conditions. The most significant levels of control of PPNs have been observed against RKNs^2^. The application of EPN infective juveniles (IJs) from different strains has significantly controlled *Meloidogyne* spp., both in the number of eggs^27^, egg masses^24^, and the infectivity of J2s inside root matrix^28^. Furthermore, the use of symbiotic bacteria and/or their metabolites alone significantly reduced RKNs J2s in vitro^29,30^ as well as decreased host infectivity in greenhouse conditions^24,31^. In a study carried out by Vyas et al.^31^, indicated that the level of control was reported to be comparable to some of the chemical treatments used. In addition, Caccia et al.^25^ reported a significant nematicidal effect of three Argentinean EPN isolates against *M. hapla* using the bacterial supernatant of *Photorhabdus luminescens* and *Xenorhabdus* spp.

Recently, several Moroccan strains of *S. feltiae* and *H. bacteriophora* were isolated by Benseddik et al.^32^. However, their efficacy has not been yet evaluated against *Meloidogyne* species. Therefore, the main objective of this study is to investigate the antagonistic activity of native EPN strains against *M. javanica* under greenhouse conditions in Morocco.

## Results

### Antagonistic activity of Moroccan EPNs toward *M. javanica*

**Figure 1:**
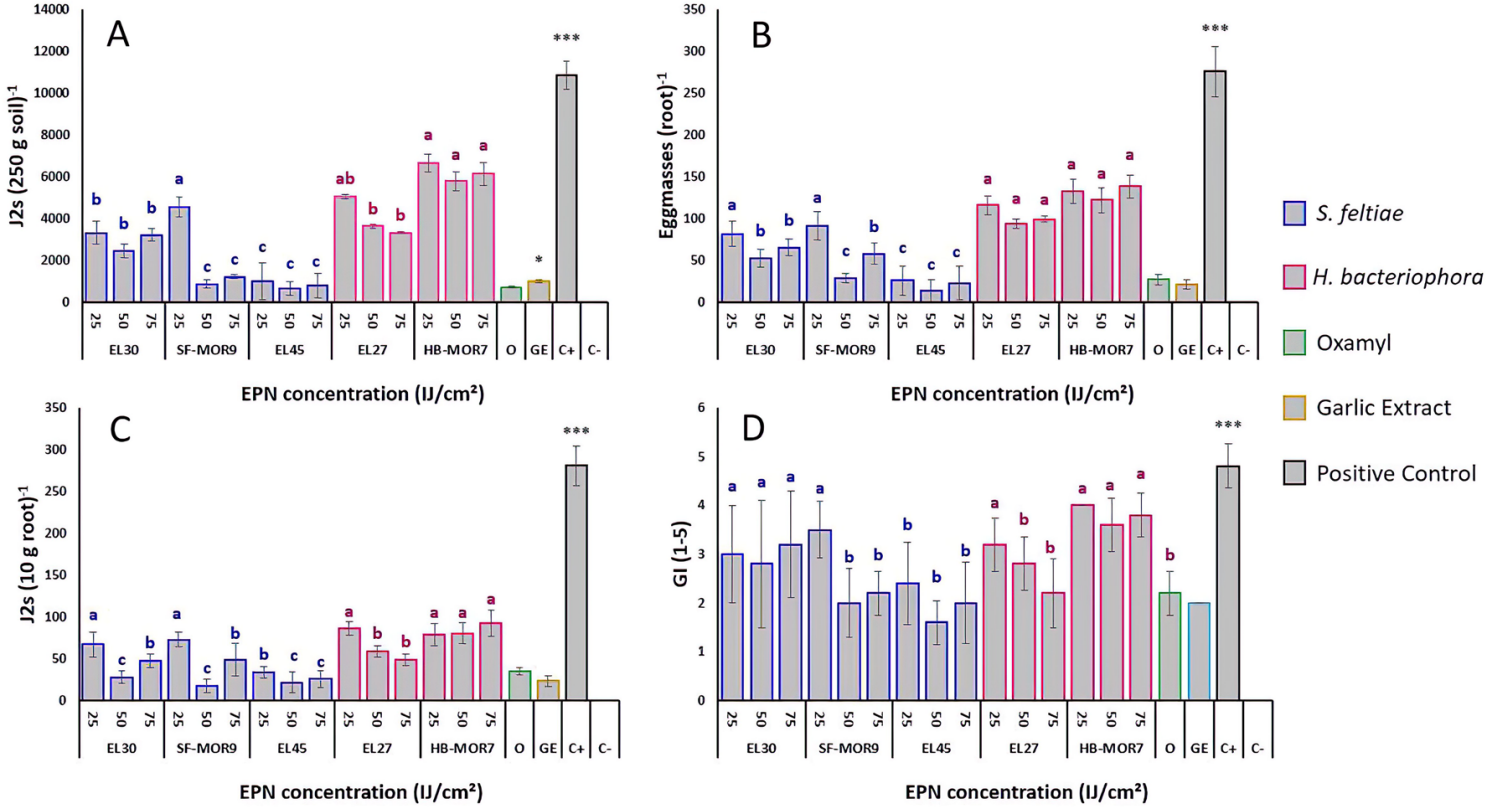
Effect of EPNs (*Steinernema* sp.,* S. feltiae* and *H. bacteriophora*) against *Meloidogyne javanica*. (**A**) Number of J2s per 250 g of soil. (**B**) Number of egg masses per root system. (**C**) Number of J2s per 20 g of root. (**D**) *M. javanica* galling index. Letters represent homogeneous groups based on protected least significant difference test (LSD) for each variable at (*P* < 0.05). Error lines on the bars represent the standard error.

The influence of the EPNs isolates on the nematological parameters is shown in Fig. 1. The number of *M. javanica* infective juveniles (J2s) in the soil was significantly reduced across the different treatments when compared to the positive control. *Steinernema* strains significantly reduced J2s densities per 250 g of soil (Fig. 1A) and per 10 g of roots (Fig. 1C) compared to *H. bacteriophora* strains. For both parameters, *S. feltiae* (EL45) strain reduced J2s densities in the soil by 95% and in the root by 90% (*F*_*index*_ = 36.4; df = 2; *P* < 0.05). SF-MOR9 strain caused significant reductions in J2s numbers when applied at 50 and 75 IJ cm^−2^ of soil. This reduction was more or less similar to the effect obtained by Oxamyl and Garlic extract treatments. On the other hand, *H. bacteriophora* strains (EL27 and HB-MOR7) demonstrated less antagonistic activity toward J2s densities in the soil and root system when compared to the positive control (*F*_*index*_ = 22.4; df = 1; *P* < 0.05). The egg-masses formation was also decreased by EPN application at both doses (Fig. 1B).

EL45 strain was significantly more efficient when compared to the other *Steinernema* strains (*F*_*index*_ = 33.8; df = 2; *P* < 0.05), while no significant difference was observed between *H. bacteriophora* strains (*F*_*index*_ = 15.3; df = 1; *P* > 0.05). The lowest galling index values (2–2.5) were observed when applying EL45 and the SF-MOR9 strains (*F*_*index*_ = 27.5; df = 2; *P* > 0.05) at 50 and 75 IJ cm^−2^. In addition, these doses were most effective when using *H. bacteriophora* strain (EL27) (*F*_*index*_ = 21.7; df = 1; *P* < 0.05) compared to the positive control (Fig. 1D), even though less effective than the other strains used.

**Figure 2:**
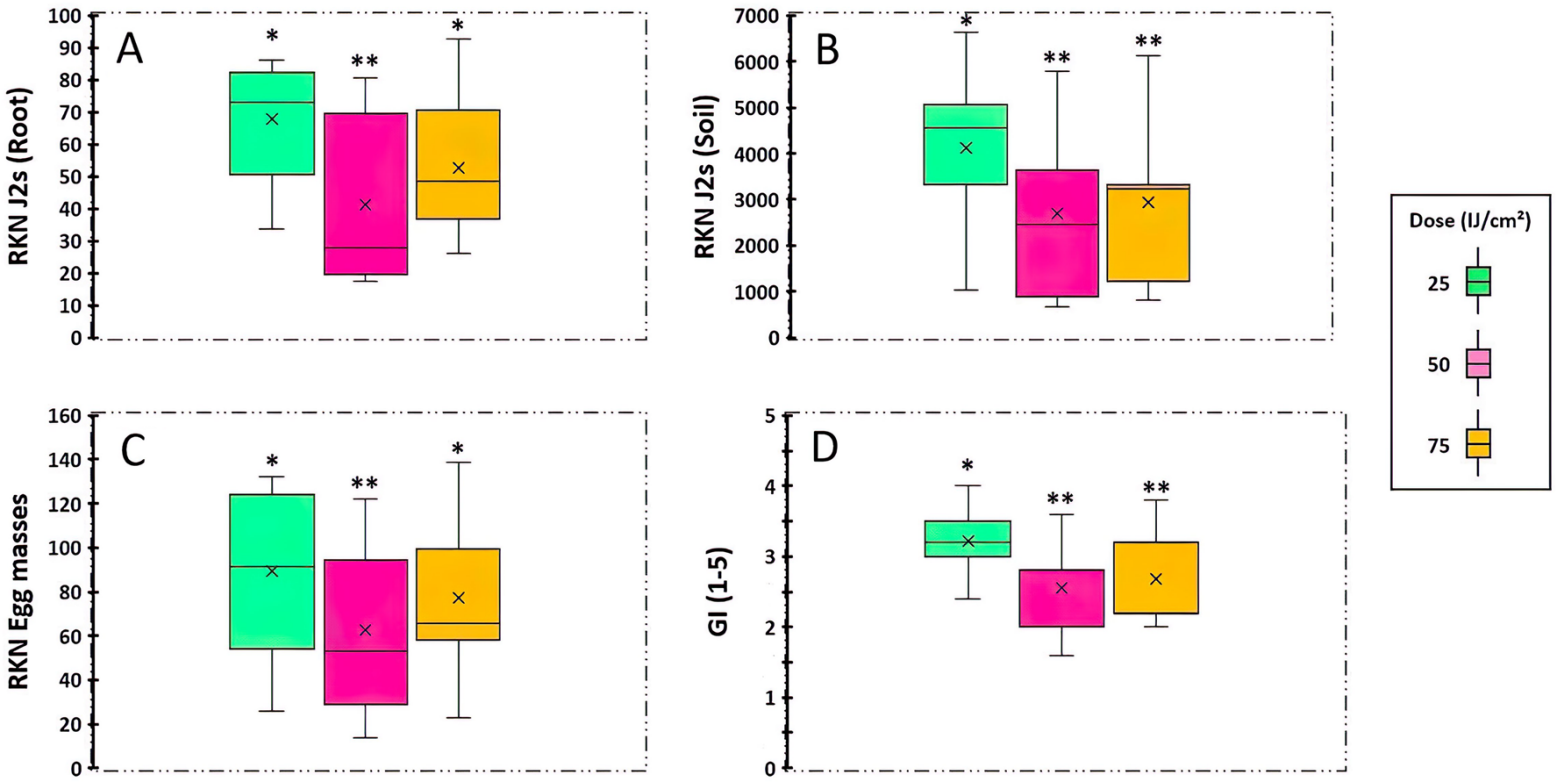
Effect of EPN dose on *M. javanica.* (**A**) Infective juveniles (J2s) per root system. (**B**) Infective juveniles (J2s) in soil matrix. (**C**) *M. javanica* egg-masses. (**D**) *M. javanica* galling index. Stars represent significant differences obtained according to the protected least significant difference test (LSD) for each variable at (*P* < 0.05).

The EPN dose applied against *M. javanica* was assessed across all the nematological parameters (Fig. 2). The number of J2s in both soil and root substrates were significantly reduced when applying EPN at rates of 25 and 75 IJ cm^−2^ (*F*_*index*_ = 42.1; df = 2; *P* < 0.05) compared to the positive control (Fig. 2A,B). The same trend was observed with the number of egg masses (*F*_*index*_ = 36.6; df = 2; *P* < 0.05) (Fig. 2C) and the galling index (*F*_*index*_ = 22.5; df = 2; *P* < 0.05) (Fig. 2D).

**Figure 3:**
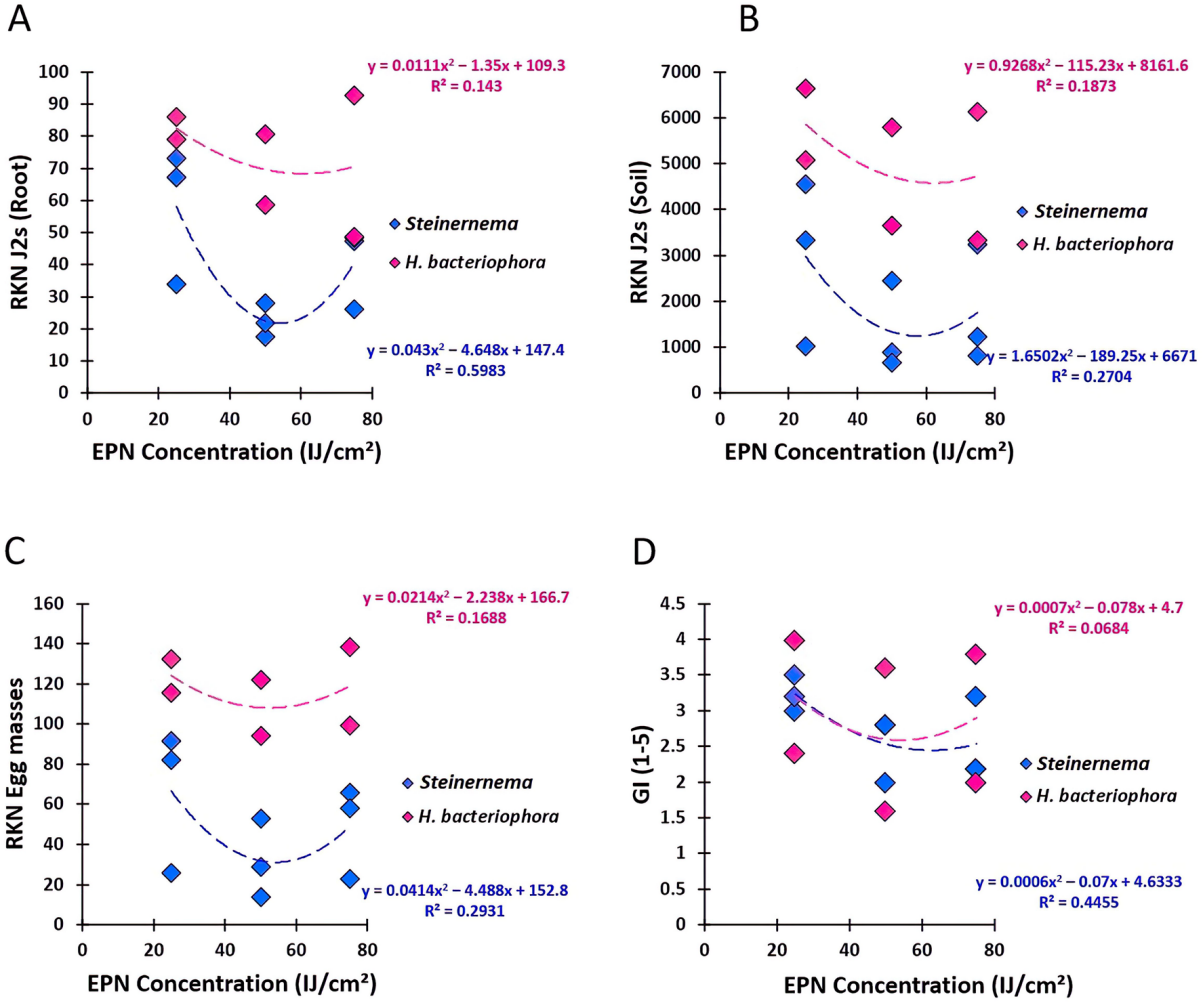
Polynomial regression analysis showed the relationship between *M. javanica* parameters and different concentrations of EPN (*Steinernema* sp*.*,* S. feltiae* and *H. bacteriophora*). (**A**) regression EPN-J2s per root system. (**B**) regression EPN-J2s in soil matrix. (**C**) regression EPN-Egg-masses. (**C**) regression EPN-Galling index. Values of R^2^ for *Steinernema* strains were significant at *P* < 0.05.

To confirm the relationship between *M. javanica* parameters and EPN concentrations, polynomial regression analyses were performed (Fig. 3). For the J2s densities, significant regression models were obtained for *Steinernema* strains in both soil and root matrices (R^2^ = 0.59 and R^2^ = 0.27; *P* < 0.05), respectively, (Fig. 3A,B). *Heterorhabditis bacteriophora* strains were not significantly affiliated with the applied doses (R^2^ = 0.14 and R^2^ = 0.18; *P* > 0.05), respectively. Similarly, the same findings were obtained between EPNs and the number of egg masses (Fig. 3C), and the galling index (Fig. 3D).

### The EPNs effect on plant growth parameter co-treated with *M. javanica*

**Figure 4:**
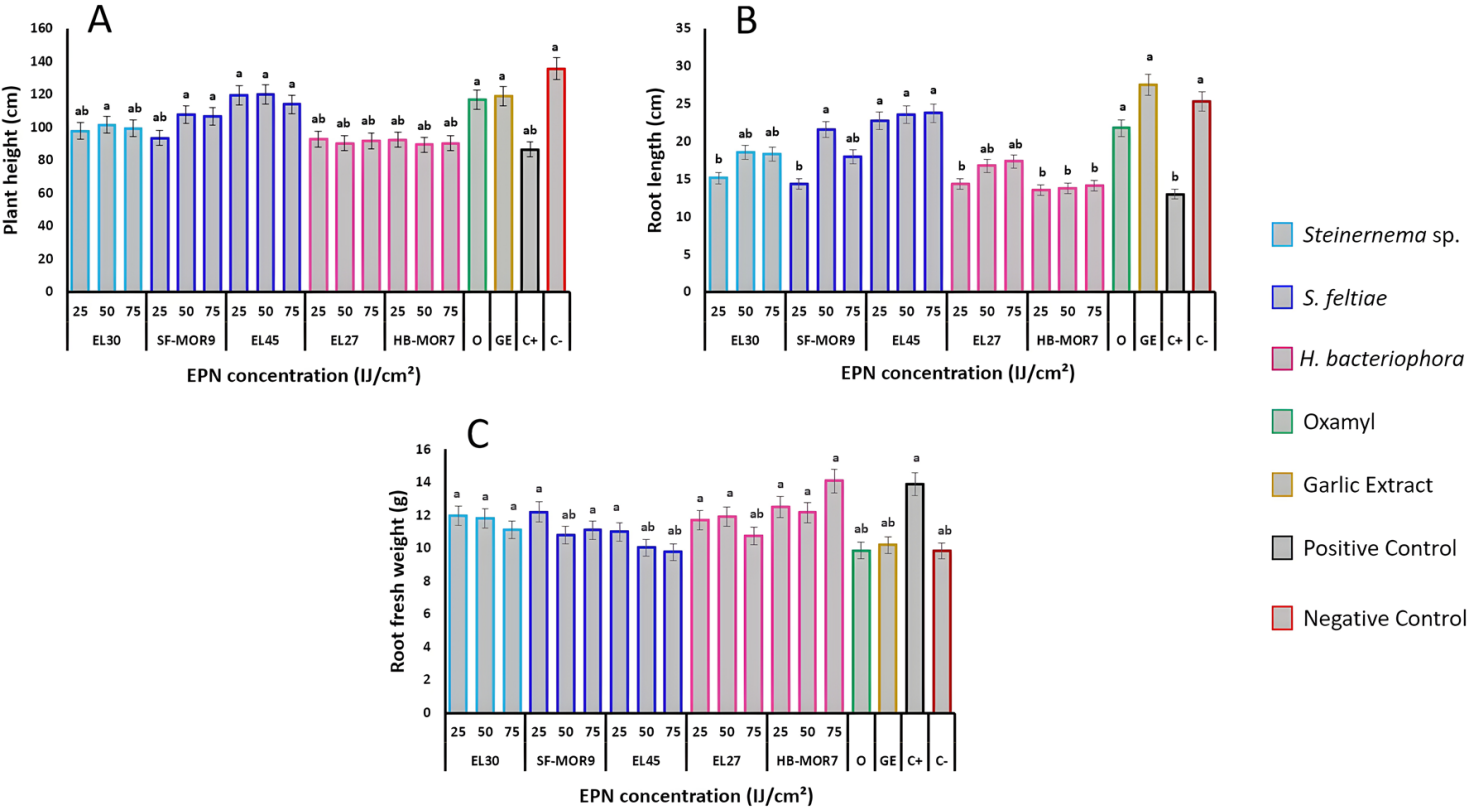
Effect of EPNs on plant growth parameters. (**A**) Plant height. (**B**) Root length. (**C**) Root fresh weight. Values represent the mean + standard error (SE); Letters represent homogeneous groups based on protected least significant difference test (LSD) for each variable at (*P* < 0.05).

EPN strains led to significant differences in plant height (*F*_*index*_ = 78.5; df = 9; *P* < 0.05) (Fig. 4). The greatest increases in plant height were observed when *S. feltiae* strain EL45 applied at 50 IJ cm^−2^ (120.2 ± 5.54 cm), this increase was similar to those recorded by the garlic extract and Oxamyl product (Fig. 4A). On the other hand, *H. bacteriophora* strains (EL27 and HB-MOR7) gave the lowest plant height values (90.04 ± 6.10 cm) but still slightly more than the positive control (86.8 ± 4.32 cm). Root length was also significantly affected by EPN strains (*F*_*index*_ = 56.4; df = 9; *P* < 0.05), with the strain EL45 effectively promoting root growth at every dose applied (23.8 ± 3.70 cm), when compared to the reference controls (Fig. 4B). An opposite trend was observed with *H. bacteriophora* strains (EL27 and HB-MOR7) with a maximum rate of 17.4 ± 2.60 cm in root growth. Root fresh weights were more pronounced with *H. bacteriophora* strain (HB-MOR7) that gave the highest values (14.08 ± 0.87 g) compared to the *S. feltiae* strains (*F*_*index*_ = 19.34; df = 9; *P* < 0.05) (Fig. 4C). The *Steinernema* strain EL30 was the most effective treatment in increasing root growth compared to the positive control.

## Discussion

The interaction between PPNs and EPNs resulted in distinct antagonistic patterns that could have implications for implementation in vegetable production and attempts to reduce the use of chemical methods. In addition, EPNs are commercially available for the management of various insect pests^33^ and could be used simultaneously for nematode and insect pest management. In this study, the antagonistic activity of five native EPNs (*Steinernema* sp., *S. feltiae,* and *H. bacteriophora*) were assessed against the RKN, *M. javanica* in tomato plants under greenhouse conditions. Both nematode infection and development, as well as their effects on tomato growth parameters, were measured over a period of 2 months. The J2 *M. javanica* densities in the soil and root were moderate to highly affected by the antagonistic effects of the different EPN treatments applied to the soil. All EPN treatments were able to reduce nematode final population densities in both the soil and root matrices when compared to the positive control. In addition, the EPNs strains decreased both egg-mass number and gall formation. *Steinernema feltiae* strains EL45 and MOR9 were significantly more effective in reducing nematode impact when compared to *H. bacteriophora* strains and the other strain of *Steinernema* sp. in which different mechanisms could have interfered. To our knowledge, this is the first investigation of the applicability of Moroccan EPNs to control *M. javanica* in tomato plants and confirms the observations made by others regarding their antagonistic interaction as mentioned in the introduction.

Previous studies indicated that the direct application of EPN IJs has shown an antagonistic effect on different PPN species^28,30,34,35^ Which are in a agreement with our findings in the current study. The observed antagonism in our study could be due either to the competitive patterns (most likely over space) between both nematode trophic levels or to the production of special chemical substances that could diverge the harmful effects of PPN. However, EPNs may not be active against all PPNs and depends on the species and crop aspects^26^. Thus, a decrease in symptoms may not always lead to significant control efficiency, especially under field conditons^36,37^. Our experiment depicted the effect of *S. feltiae* strains on RKN indices to be significantly reduced compared to the positive control confirming their potential efficacy, but not to the level of Oxamyl and garlic extract products. In addition, the sensitivity of nematode parameters could be the main reason behind this antagonistic response. Similarly, in cucumber plants, Sayedain et al*.*^38^ reported that *S. carpocapsae* and *H. bacteriophora* were shown to decrease all the pathogenicity indices (number of galls, eggs, and egg masses) of *M. javanica* in both growth chamber and greenhouse conditions. The same findings were obtained by Fallon et al.^36^, indicating that applying both *S. feltiae* MG-14 and *S. feltiae* SN strains significantly minimized *M. javanica* invasion on soybean 3 days after treatment but did not affect *M. javanica* egg formation in tomato plants after 30 days. Pérez and Lewis^27^ had applied *H. bacteriophora* and *S. feltiae* (25 IJs cm^−2^) before and after inoculation of *M. incognita*. They observed that these EPNs were able to inhibit the penetration of this RKN and decrease the production of eggs on tomato plants. However, the effect was not proficient enough against *M. javanica*^27^. In the same context, *S. feltiae* was confirmed to be ineffective against *M. javanica* on cucumber^38^ and the authors emphasized that adopting other *Steinernema* strains (e.g., *S. riobrave* and *S. carpocapsae*) may induce stronger antagonistic effects against this parasitic nematode.

*Heterorhabditis bacteriophora* strains (EL27 and HB-MOR7) applied in aqueous suspension reduced *M. javanica* infection indices compared to the positive control. However, the reduction in nematodes parameters by *H. bacteriophora* was inconsistent when compared to *S. feltiae.* Other studies are in agreement with our results regarding to *H. bacteriophora* effectiveness against other RKN species. For instance, Smitley et al.^39^ mentioned that applying *H. bacteriophora* did not reduce *M. rusticum* densities in turf, while Pérez and Lewis^27^ noticed that the IJs of *H. bacteriophora* did not produce an antagonism towards penetration aspects of *M. incognita* and *M. hapla* in peanut (*Arachis hypogaea*) unlike *S. feltiae*. This divergence between EPN genera was explained by the fact that *S. feltiae* could enter the roots releasing their bacteria better than *H. bacteriophora*, causing a more consistent effect. On the other hand, Kepenekci et al.^24^ refuted the idea behind diverged virulence attributes of both EPNs, as they potentially reduced egg masses of *Meloidogyne* spp. in tomatoes. Regarding the interaction between EPNs and the tomato plant in reducing the invasion of PPNs, the infection behavior of *M. javanica* towards the root system needs to be examined and explained. The hijacking of new roots above the root cap by freshly hatched J2s followed by EPNs attraction to the root tips could occur and cause antagonism^37^ and this behavior could cause a blockage of the penetration process of *M. javanica.* In addition, *Steinernema* spp. can enter the root system and release their embodied bacteria^27^, making them able to hurtle nematode life cycle.

The antagonistic effects of EPNs toward *Meloidogyne* spp. are closely associated with the application time frame, inoculum density, host plant, and the species of both the PPN and EPN^40^. In our study, both 50 and 75 IJs cm^−2^ EPN doses were shown to be effective in the control of *M. javanica* compared to the positive control. Sayedain et al.^38^ found that applying densities of 125 IJs cm^−2^ (19.1 IJs cm^−3^) significantly increased the biocontrol of *M. javanica*. Similarly, Pérez and Lewis^27^ confirmed that using 125 IJs cm^−2^ of *H. bacteriophora* co-inoculated with *M. hapla* in peanut reduced the egg production, while the same dose of *S. riobrave* did not inhibit the J2s of *M. hapla*. The observed antagonistic activity of EPNs against *M. javanica* in this study might be highly related to allelochemicals and ammonium production by the associated symbiotic bacteria^41^, plants systemic resistance^42^, competitive patterns EPN-RKN, and EPNs attraction toward exudates emitted by the plant root system^40^. In our study, the reduction of *M. javanica* by *S. feltiae* strains (EL45 and SF-MOR9) may have been due to metabolites produced by its mutualistic bacteria (*Xenorhabdus* spp.). Therefore, the interactions involved are complex and due to the interference of a tripartite system (host plant, PPN, and EPN)^43^.

In the current study, the tested EPNs has significant role on plants' growth parameters. That the results indicated that the *S. feltiae* strain EL45 caused significant increase in both plant height and root length when compared to the positive control, and this increase was similar to that obtained by the Oxamyl and garlic extract. while the *H. bacteriophora* strains EL27 and HB-MOR7 have only enhanced tomato fresh root weight. Conversely, Sayedain et al.^38^ reported significant increases in root fresh weight of cucumber plants when *S. carpocapsae* was applied. However, previous studies have reported inconsistent effects of different EPNs IJs on plant dry weight^36,44^ as well as their potential for promoting plant biomass^29,45^. In our study, this effect may imply the nullifying characteristics of EPN species towards PPN occurrence in the soil and thus investing in plants' growth and development.

In conclusion, this study provides insights into the practical usage of EPNs as biological control agents against the root-knot nematode *M. javanica* on tomato plants. Our results demonstrated that inoculation of *S. feltiae* strains (EL45 and SF-MOR9) with *M. javanica* J2s leads to significant levels of antagonistic activity. *Heterorhabditis bacteriophora* strains (HB-MOR7 and EL27) gave inconsistent results in terms of *M. javanica* infection and plant growth parameters. Further studies are required to evaluate the effectiveness of these endemic EPN strains in commercial greenhouses and optimize their inputs on plants yielding aspects. The interaction between EPNs and PPN needs to be further studied to determine if one EPN inoculation is sufficient or multiple treatments over a longer period of time are required. In addition, further studies are needed to determine whether the EPNs alone are responsible for control, or the bacteria *Photorhabdus luminescens* and *Xenorhabdus* spp. which are released into the root tissue are involved.

## Materials and methods

### Preparation of *Meloidogyne javanica*

*Meloidogyne javanica* pure population was maintained by isolating single egg masses from tomatoes in the Souss-Massa region, hatched as indicated below, and thereafter maintained on tomato (*Solanum lycopersicum* L.) in the greenhouse of the biotechnology research unit at INRA-Rabat. The commercial tomato seeds (Zayda; Rijk Zwaan) were purchased, and experiments were carried out according to the guidelines and regulations of the Moroccan Agriculture Ministry. Eggs of *M. javanica* were extracted from tomato roots infected with the nematode using the sodium hypochlorite method of Hussey and Barker^46^. The resulting egg suspension was incubated for 3 days at 25 °C to allow second-stage juveniles (J2) hatching. Newly hatched J2s that were not more than 48 h old were used in the experiments.

### Source of entomopathogenic nematodes

The EPNs strains isolated from soil in Morocco were evaluated against *M. javanica* as listed in (Table 1). These EPNs included: two isolates of *Steinernema feltiae*, two isolates of *Heterorhabditis bacteriophora,* and one isolate of *Steinernema* sp. Each EPN strain was reared in vivo at 25 °C on the last instar larvae of *Galleria mellonella* Linnaeus (Lepidoptera : Pyralidae), according to Kaya and Stock^47^. Dead larvae of *G. mellonella* were placed on white trap^48^ and infective juveniles (IJs) were harvested and stored at 8 °C in a 0.5-L container filled with distilled water. The viability of nematodes was checked by observing the movement of IJs under stereomicroscope before use^49^.

**Table 1:** Native EPN strains of different species accessions used against *Meloidogyne javanica* under greenhouse experiments.

Nematode species	Isolate	Genbank accession no	Origin	Vegetation
*Steinernema feltiae*	SF-MOR9	MN749619	El Jorf/Fezna	Date palms (*Phoenix dactylife*ra L.)
	EL45	MZ265250	INRA-Rabat	Citrus trees
*Heterorhabditis bacteriophora*	EL27	MZ265242	Gharb	Olive trees (*Olea europaea* L.)
	HB-MOR7	MN420696	El Fouarate	Plum trees (*Prunus* sp.)
*Steinernema* sp.	EL30	MZ265229	INRA-Rabat	Fig trees (*Ficus carica*)

### Greenhouse pot experiments

Evaluation of EPNs effectiveness against *M. javanica* was performed in pot experiments under greenhouse conditions at the Biotechnology Research Unit (INRA, Rabat, Morocco). Tomato seedlings with three pairs of leaves were transplanted individually into 3.5-L plastic pots (16 cm-upper diameter and 20 cm height) filled with autoclaved soil mixture (84% sand, 12% silt, 4% clay). After five days, *M. javanica* and EPN treatments were applied simultaneously. Each tomato seedling was inoculated with 4500 freshly hatched J2s of *M. javanica* suspended in 20 mL water. The J2s were applied by flooding the solution on the surface of the pot. Infective juveniles of each EPN strain were applied at the rates of 25 IJs cm^−2^ (5024 IJs/pot), 50 IJs cm^−2^ (10,048 IJs/pot), and 75 IJs cm^−2^ (15,072 IJs/pot) per each tomato seedling. In the same manner, the plants served as positive controls were inoculated with J2s of *M. javanica* and sterile distilled water was used instead of EPN. The negative control plants were only received distilled water. Oxamyl and Garlic extract treatments were used as reference products for effect comparison. The chemical pesticide (Oxamyl) was applied to tomato seedlings one day after nematode inoculation at a rate of 0.4 mL/plant. Garlic extract was incorporated in the top layer of the substrate at a rate of 1.4 g per pot*,* just after transplantation, followed by irrigation to initiate the dissolution of granules. The experiment was arranged in a completely randomized design with five replications per EPN treatment and repeated twice for data validation.

### Data collection

Two months after *M. javanica* inoculation, experiments were terminated and tomato plants were uprooted and gently washed. Nematological parameters (i.e., number of J2s *M. javanica*, egg masses, and gall index) and plant growth parameters (i.e., plant height, root length, and root fresh weight) were determined for each plant. To estimate the final population of J2s *M. javanica,* soil from each pot was thoroughly mixed and a 250 g subsample was used for nematode extraction as per Baermann tray method^50^. Roots were stained in Phloxine B for 20 min^51^ (Daykin and Hussey, 1985) and then the total number of egg masses were counted per root system under a stereomicroscope. The root of each sample was gently washed in tap water to free adhered soil particles, cut into pieces (ca 0.5 cm), and then J2s *M. javanica* were extracted from 10 g subsample using Baermann tray method^50^. Root galling caused by *M. javanica* was indexed on each tomato root using a 0 to 5 scale^52^ as follows: 0 = no galls, 1 = 1–2 galls, 2 = 3–10 galls, 3 = 11–30 galls, 4 = 31–100 galls and 5 =  > 100 galls.

### Statistical analysis

*Meloidogyne javanica* and plant parameters were subjected to ANOVA procedure using the XLSTAT software, ver. 2016.02.28451 (Addinsoft, New York, USA). Datasets were normalized using the Anderson–Darling normality test^53^. Each trial was independently repeated twice. A two-way ANOVA test was performed to examine sources of variation in the observed variables. Significant differences among variables were tested using protected least significant difference and Fisher's protected least significant difference (LSD) test at *P* < 0.05. Differences obtained at levels of *P* < 0.05 were considered significant. Polynomial regression analysis was established to describe the relationship between nematode parameters and the applied EPN concentrations. All multivariate analyses were performed using R 3.4.3 software^54^.

### Ethical approval

This article does not contain any studies with human participants or animals performed by any of the authors.
